# Cutoff Values of Aldosterone and the Aldosterone–Renin Ratio for Predicting Primary Aldosteronism in Patients with Resistant Hypertension: A Real-Life Study

**DOI:** 10.3390/jcdd11100299

**Published:** 2024-09-27

**Authors:** João Vicente da Silveira, Carine Sangaleti, Cleber Camacho, Ana Alice Wolf Maciel, Maria Claudia Irigoyen, Thiago Macedo, José Jayme G. De Lima, Luciano F. Drager, Luiz Aparecido Bortolotto, Heno Ferreira Lopes, Madson Q. Almeida, Brent M. Egan, Fernanda Marciano Consolim-Colombo

**Affiliations:** 1 Medical School, Universidade Nove de Julho—Uninove, São Paulo 04101-200, Brazil; joao.silveira@uol.com.br (J.V.d.S.); camacho@uninove.br (C.C.); hipfernada@incor.usp.br (F.M.C.-C.); 2 Cardiology Department, Unidade de Hipertensão, Instituto do Coração—Universidade de São Paulo—HC-FMUSP, São Paulo 05403-000, Brazil; hipirigoyen@gmail.com (M.C.I.); thiago.cardio@gmail.com (T.M.); jose.lima@incor.usp.br (J.J.G.D.L.); luciano.drager@incor.usp.br (L.F.D.); hipbortolotto@gmail.com (L.A.B.); 3 Nurse Department, Universidade Estadual do Centro-Oeste—Unicentro, Guarapuava 85040-167, Brazil; carineteles@unicentro.br; 4 Unidade de Hipertensão, Disciplina de Nefrologia—Universidade de São Paulo—HC-FMUSP, São Paulo 05403-010, Brazil; anaalice.wolf@gmail.com; 5 Laboratório de Endocrinologia Molecular e Celular LIM/25, Disciplina de Endocrinologia e Metabologia, Hospital das Clínicas, Faculdade de Medicina da Universidade de São Paulo (FMUSP), São Paulo 05403-010, Brazil; madsonalmeida@gmail.com; 6 Cardiovascular Health, American Medical Association, Medical University of South Carolina, Greenville, SC 29425, USA; brent.egan@ama-assn.org; 7 Medicine and Nursing, Medical University of South Carolina, Charleston, SC 29425, USA

**Keywords:** primary aldosteronism, secondary hypertension, aldosterone–renin ratio, aldosterone, apparently resistant hypertension, reference value

## Abstract

Primary aldosteronism (PA) is commonly associated with resistant hypertension. Biochemical tests can be clinically useful in the screening and diagnosis of primary aldosteronism. This study aimed to identify the cutoff values of aldosterone levels (A) and the aldosterone–renin ratio (ARR) for an accurate prediction of PA in patients with apparent resistant hypertension in a real-life scenario. This database-based study included a historical cohort of male and female patients with apparent resistant hypertension, aged 18 years or older and surveyed for PA in a specialized center from 2008 to 2018. Aldosterone and plasma renin activity (PRA) or the plasma renin concentration (PRC) were measured in the treated hypertensive patients. The patients with positive screening results were subsequently referred to the endocrinology department for confirmatory tests. The patients with confirmed PA were included in the case group, and the others remained as controls. Receiver-operating characteristic (ROC) curves were used to identify the cutoff points for aldosterone and the ARR, thereby analyzing their sensitivity and specificity for confirmed PA. Among the 3464 patients (59 ± 13 years old, 41% male) who had apparent resistance hypertension screened, PA was confirmed in 276 individuals (8%). A ≥ 16.95 ng/dL (95% CI: 0.908–0.933) had an odds ratio of 6.24 for PA, while A/PRA ≥ 29.88 (95% CI: 0.942–0.984) or an A/PRC ≥ 2.44 (95% CI: 0.978–0.990) had an odds ratio of 216.17 for PA diagnoses. Our findings suggest that a positive PA screening with aldosterone ≥ 17 ng/dL associated with A/PRA ≥ 29.88 or an A/PRC ratio of ≥2.44 should be sufficient to confirm the diagnosis of PA without confirmatory testing.

## 1. Introduction

Recent guidelines define primary aldosteronism (PA) as a group of disorders in which aldosterone production is inappropriately high and relatively autonomous from the physiological control of the renin–angiotensin system [1,2,3,4,5,6]. Such inappropriate aldosterone production causes sodium retention and potassium excretion, hypertension, cardiovascular damage, and the suppression of plasma renin [7,8].

Initially, PA was considered a rare form of secondary hypertension. However, cross-sectional and prospective studies report PA in 3 to 10% of hypertensive patients in general, reaching a higher prevalence in particular settings such as resistant hypertension (patients with a blood pressure greater than 140/90 mmHg using three antihypertensive drugs, including diuretics) [9,10,11,12]. There are two main etiologies of PA: aldosterone-producing adenoma, as described by Cohn in 1955, which accounts for approximately 30–40% of cases, and idiopathic aldosteronism or bilateral adrenal hyperplasia, which is detected in approximately 60–70% of cases [13,14]. Germline mutations affecting genes in aldosterone synthesis may generate a small fraction of rare cases of PA [15]. The early detection of PA enables targeted treatment, enhances blood pressure control rates, and reduces associated cardiovascular morbidity and mortality. Unfortunately, recent studies indicate insufficient rates of PA screening among hypertensive patients, including those with apparent treatment-resistant hypertension (only 2%) [16,17,18]. Screening for PA aims to distinguish between this secondary cause of hypertension and essential (primary) hypertension [18].

A high plasma aldosterone concentration is a hallmark of PA, while the most reliable tool for PA screening is a high aldosterone–renin ratio (ARR) [19]. Current guidelines suggest that aldosterone levels surpassing 12–15 ng/dL are a reliable indicator of PA [1,6]. Plasma renin activity (PRA) [20,21] or the direct plasma renin concentration (PRC) [22,23] are considered the main methods for conducting screening tests in patients with PA. Nevertheless, the consensus on the definitive cutoff values for the ARR must be improved, as they may vary depending on several factors, including the use of medications [22,23,24,25]. The actual advised cutoff for the A/PRA ratio is 20 to 30 (ng/d)/(ng/mL/h). Regarding the A/PRC ratio, the guidelines suggest a cutoff of 2.0 to 2.5 ng/dl, but several publications consider a more extensive range of values from 1.0 to 5.7 (ng/dL)/(µU/mL) [26,27,28].

After a positive screening, subsequent evaluations are mandatory to confirm PA and its etiology. These assessments include protocols to evaluate the adrenal gland at functional, structural, and even molecular levels and require specialized centers and economic resources [7,8,14,15]. In this scenario, the delayed detection of PA negatively impacts the prognosis of the patients [18,19].

In the present study, we aimed to investigate the cutoff values of the aldosterone level, the A/PRA ratio, and the A/PRC ratio that best predict PA in a large cohort of patients who were referred to a tertiary hospital for the evaluation of treatment-resistant hypertension. This information would reinforce the importance of PA screening and reduce delays in the diagnosis and appropriate treatment of PA in this high-risk population.

## 2. Material and Methods

### 2.1. Study Population

This study analyzed medical records from hypertensive individuals treated at the University of São Paulo’s Heart Institute/Clinical Hospital between 2008 and 2018, with ethical approval by the Institution`s Human Research Committee of Hospital das Clinicas—Sao Paulo University (Protocol SDC-COP 105.19.011, approved on 30 March 2019). Adult patients with treatment-resistant severe hypertension underwent aldosterone and renin testing while on antihypertensive medication. However, the patients were instructed to discontinue mineralocorticoid receptor antagonists four weeks prior to the test, in accordance with guidelines [1,2,3,4,5,6]. We removed duplicates and incomplete records, and excluded patients with chronic kidney disease, dialysis, or severe systemic diseases. The data collected included demographics, blood pressure measures, and laboratory test results, such as creatinine, electrolytes, glucose, and HbA1C. The estimated glomerular filtration rate (eGFR) was determined using appropriate formulas, and 24 h urine sodium was measured.

Morning venous blood samples were collected after the patients had been out of bed for at least 2 h and typically after they had been seated for 5–15 min for aldosterone and renin assays. In 2012, the assay method transitioned from radioimmunoassay to chemiluminescence-based methods [7,25].

We set the aldosterone thresholds at 10 ng/dL for proceeding with the PA investigation, adjusting the PRA and PRC values to reduce false positives. An A/PRA ratio ≥ 30 ng/mL/h and an A/PRC ratio ≥ 2.5 ng/dL indicated positive screening.

**Figure 1:**
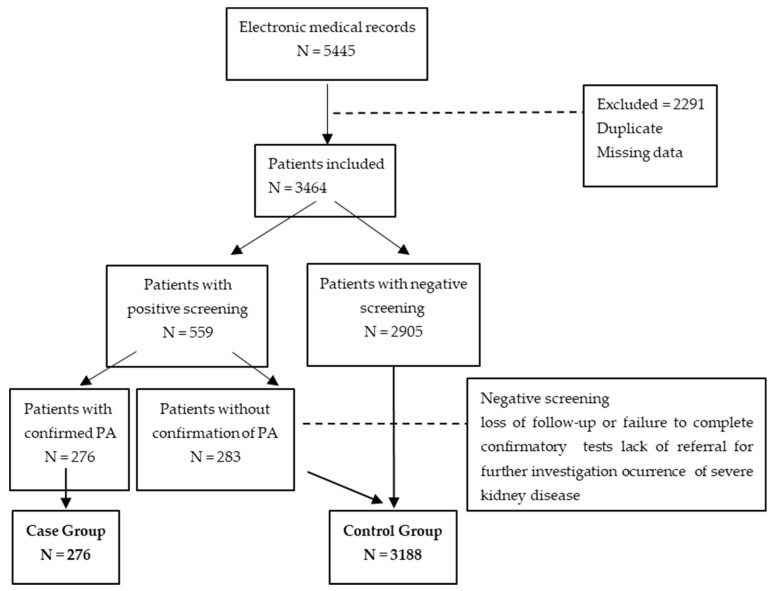
Flow chart of the protocol.

Positive screening led to referral for further PA confirmation and etiology assessment at the Endocrine Division, which included functional tests and adrenal imaging [7,25]. Only the patients who completed the diagnostic process and received PA treatment were included in the case group, and the rest served as controls (Figure 1).

### 2.2. Statistical Analysis

The descriptive statistics included frequencies and summary statistics, and the data are expressed as the mean ± SD. Chi-square tests were used to analyze the categorical variables, while nonparametric tests (Mann–Whitney or Fisher’s Exact tests) were used for the data that were not normally distributed. ROC curves determined the cutoffs for aldosterone and the ARR, while logistic regressions evaluated the predictors of PA. The Hosmer and Lemeshow test assessed the model fit, with the CHAID (Chi-Square Interaction Detector) algorithm aiding in the multivariate analysis. The Bonferroni correction was applied, and a 5% significance level was used for all the tests. The analyses were conducted using IBM SPSS Software, version 25.0 (Armonk, NY, USA).

## 3. Results

The database initially held 5445 records; after exclusions, 3464 the patients remained for the analysis. Of these, 559 (16.1%) had a positive PA screen with aldosterone >10 ng/dL and a high ARR; 276 were diagnosed with PA and constituted the case group, while the others were considered as controls.

Table 1 describes the study cohort, showing an average age of 59 years, an overweight status, and a high systolic blood pressure, despite treatment. Serum potassium was normal on average, while creatinine and HbA1c were slightly elevated. Most the patients (70%) had the PRA measured, with an average A/APR ratio of 17.4 ± 3.7 and a median of 8.0. The PRC was available for 30%, yielding an average A/PRC ratio of 1.9 ± 0.4). The study population was predominantly female (60%). Compared to the men, the women had higher blood pressure and HbA1C values, while the men had higher creatinine levels, a lower eGFR, and higher urinary sodium excretion.

Table 2 indicates that using an A/PRA ratio ≥ 30, 14.7% of the patients screened positively, while with an A/PRC ratio ≥ 2.0, the value was 19.5%. The men had a higher positive screening prevalence. Of the patients with positive screens, 347 were further investigated at the Endocrine Service, 276 had confirmed PA, and 71 did not. The case group (n = 276) was used for the statistical analysis, with the remainder serving as controls (n = 3188).

The ROC curve analysis (Table 3) showed that an aldosterone level of ≥16.95 ng/dL had a sensitivity of 84.8% and a specificity of 83.4%, but the accuracy was 8.5%. An A/PRA ratio ≥ 29.88 and an A/PRC ratio ≥ 2.44 demonstrated high accuracy (90.4% and 93.8%, respectively), sensitivity, and specificity, with high positive and negative predictive values.

The cases (Table 4) were younger and had lower blood pressures and potassium levels compared to the controls. As expected, the mean aldosterone level was higher in the case group. Additionally, the median A/PRA and A/PRC levels were notably higher in the case group than in the controls. 

Multivariate logistic regression (Table 5) showed that for every 1 mEq/L increase in potassium, the odds of PA decreased by 45%. The patients with aldosterone ≥ 16.95 ng/dL or an A/APR ≥ 29.88 or an A/PRC ≥ 2.44 were substantially more likely to have confirmed PA, highlighting the need for precise evaluation in these patients.

## 4. Discussion

Primary aldosteronism, marked by high aldosterone and suppressed renin levels, is often under-recognized, despite its prevalence of up to 20% in resistant hypertension. Our retrospective, single-center study confirmed a significantly higher likelihood of diagnosing PA with higher aldosterone levels and a higher ARR. We focused on the patients with uncontrolled hypertension who were on multiple medications, finding a mean blood pressure of almost 160/96 mmHg across the cohort. The average age was 58.9 years, with the females being slightly older, and the males showing lower glomerular filtration rates, indicating a high cardiovascular risk profile requiring diligent evaluation for secondary hypertension. Medication adjustments were avoided due to the risks involved in our severely hypertensive cohort. Our aldosterone threshold (>10 ng/dL) was lower than the guidelines suggest, minimizing the potential false negatives. Over a decade, we screened 2414 the patients with PRA and 1050 with PRC, identifying a significant PA prevalence.

Among the patients completing the diagnostic algorithm, 80% confirmed PA, with a higher-than-average incidence of adenoma (68%). Those with bilateral hyperplasia received targeted medication. The 20% false-positive rate for PA screening was lower than previously reported, suggesting our tests effectively minimized unnecessary confirmatory testing. Although in a recent review the authors suggested repeating aldosterone and renin measurements to increase the sensitivity of the ARR in diagnosing PA, in our study, only one sample proved to be sensitive for diagnosing PA [29].

The ROC curve analysis helped refine the diagnostic criteria, though a potential verification bias exists due to the absence of confirmatory tests for “negative” screening results. Our control group definition aligned with those of recent studies, which vary considerably in similar research. The aldosterone cutoff of ≥16.95 ng/dL exceeded guideline recommendations, yielding high sensitivity and specificity, but a modest positive predictive value. The A/PRA ratio threshold closely matched the upper guideline limit, with high accuracy and an excellent negative predictive value. The A/PRC ratio cutoff demonstrated high accuracy and excellent predictive values, outperforming those in the patients who ceased antihypertensives in previous studies. The multivariate analysis indicated that an aldosterone level ≥ 16.95 ng/dL and ARR values above our cutoffs substantially increased the PA risk. The cases of confirmed PA were generally younger, with lower blood pressures and potassium levels, but no single clinical or laboratory feature stood out as a definitive PA indicator. Plasma renin and aldosterone levels usually decrease with age, but renin decreases more, raising the A/R ratio in older individuals. Optimal A/PRC ratio cutoffs vary by age group: >3.7 for those ≥60 years, 2.0 for 40–59 years, and 1.0 for <40 years. Our study, with an average participant age of 60 years and a chosen A/PRC cutoff of ≥2.44, suggests that age-related adjustments may be necessary. Although the mean serum potassium was within the normal range, the lower levels identified in the case group support that normokalaemia does not rule out PA, as most of the patients would be missed. Our analysis also indicated that every 1 mEq/L increase in potassium decreased the PA odds by 45%. Hypokalemia is indeed a common feature of primary aldosteronism (PA), although it is not always present [1,2,3,4,5,6]. Studies have shown that only approximately 30–40% of patients with PA exhibit significant hypokalemia [1,2,3,4,5,6]. This study’s result demonstrated that a 1 mEq/L increase in serum potassium was associated with a 45% decrease in the odds of PA. Conversely, higher serum potassium levels may suggest a lower likelihood of PA, as the excess aldosterone would typically drive potassium levels down. The multivariate logistic regression analysis, which likely accounted for potential confounders, strengthened the validity of this association between serum potassium and the odds of PA. The aldosterone/potassium (A/K) ratio, also known as the aldosterone–potassium ratio (APR), has been proposed as a simple screening tool for PA [30]. An elevated APR suggests an increased likelihood of PA, and some studies have demonstrated its usefulness in identifying the patients who should undergo further testing for PA.

Moreover, a recent publication by Puar et al. (2020) [30] described that the aldosterone-to-lowest-potassium ratio is a convenient score to guide the clinicians of patients of various ethnicities on the probability of the primary aldosteronism subtype. The authors concluded that using the APR to identify the patients more likely to benefit from adrenal vein sampling (AVS) may be a cost-effective strategy to manage this common condition. We intend to calculate the aldosterone–potassium ratio and compare the results with the aldosterone–renin ratio (ARR) in a future publication, as it was not the main objective of the present study.

Some final considerations need to be addressed. Renin synthesis is influenced by prostaglandins and cyclooxygenase (COX) activity, with NSAIDs impacting these factors and consequently affecting renin levels [31]. Prostaglandins such as prostacyclin (PGI2) and prostaglandin E2 (PGE2) stimulate renin secretion and are synthesized by COX-1 and COX-2. NSAIDs inhibit COX activity, reducing prostaglandin synthesis and, thus, renin secretion [31]. Research indicates that COX-2 inhibitors can significantly decrease plasma renin levels and renal renin activity [32,33]. Randomized crossover studies in healthy individuals have shown that COX-2 inhibitors, like rofecoxib and meloxicam, inhibit renin release when administered with furosemide or a low-sodium diet [33]. Consequently, NSAID use can affect the interpretation of the aldosterone–renin ratio (ARR), and it is advisable to discontinue these medications before testing. In our study, we did not differentiate the data based on NSAID use, nor did we specifically screen for chronic NSAID use. Although the patients were instructed to avoid NSAIDs before the confirmatory tests, we cannot rule out short-term use at the time of screening. This represents a limitation of our study and may have influenced the results. Future research should collect detailed information on NSAID use and account for this factor in the data analysis.

### Limitations of the Study

While our study provides valuable insights into PA screening, several limitations merit consideration. The single-center, retrospective design may limit the generalizability across diverse populations. Although justified by our high-risk cohort, the absence of an antihypertensive medication washout period could have influenced the screening outcomes. Future research should stratify the results by ethnicity, age group, and medication use, including NSAIDs, to offer more nuanced insights. The potential verification bias due to the absence of confirmatory tests for negative screenings necessitates a prospective study design with universal confirmatory testing. Lastly, our study did not explore the aldosterone–potassium ratio (APR) as a screening tool, which recent studies suggest may be valuable, particularly in diverse ethnic populations.

## 5. Conclusions

In conclusion, our data suggest that aldosterone ≥ 17 ng/dL with an A/PRA ≥ 29.88 or an A/PRC ≥ 2.44 could reliably diagnose PA without additional testing.

## Figures and Tables

**Table 1:** Characteristics of all included patients and stratified by sex-based groups.

	Total	Mean ± SD	Female	Mean ± SD	Male	Mean ± SD	*p*
	Number		Number		Number		
Age (years old)	(3464)	58.90 ± 13.1	2060	59.60 ± 12.90	1404	57.80 ± 13.2	<0.001
BMI (kg/m^2^)	(3302)	29.01 ± 5.60	1965	29.89 ± 6.02	1337	29.33 ± 4.90	0.109
SBP (mmHg)	(3328)	159.58 ± 30.84	1978	160.71 ± 31.14	1350	157.92 ± 30.32	0.007
DBP (mmHg)	(3328)	95.75 ± 17.94	1978	95.59 ± 17.70	1350	95.98 ± 18.30	0.857
Creatinine (mg/dL)	(3432)	1.28 ± 1.14	2036	1.10 ± 0.87	1396	1.54 ± 1.41	<0.001
GFR (mL/min/1.73 m^2^)	(3305)	86.30 ± 38.72	1969	91.45 ± 39.86	1336	78.71 ± 35.64	<0.001
K^+^ (mEq/L)	(3259)	4.25 ± 0.57	2040	4.28 ± 0.52	1390	4.24 ± 0.58	0.857
Urinary Na^+^ (mEq/L)	(2205)	173.84 ± 82.04	1329	157.37 ± 71.97	876	198.88 ± 89.71	<0.001
HbA1c (%)	(2979)	6.51 ± 2.21	1787	6.57 ± 2.48	1192	6.42 ± 1.72	0.021
Aldosterone (ng/dL)	(3464)	13.45 ± 16.70	2060	13.13 ± 18.69	1404	13.92 ± 13.24	<0.001
PRA (ng/mL/h)	(2414)	3.88 ± 8.83	1366	2.13 ± 4.48	1048	6.15 ± 12.01	<0.001
A/PRA	(2414)	17.44 ± 36.97	1366	18.57 ± 44.29	1048	15.96 ± 24.28	<0.001
PRC (µUI/mL)	(1050)	57.74 ± 108.44	694	48.41 ± 93.07	356	75.92 ± 131.68	0.157
A/PRC	(1050)	1.87 ± 3.83	694	1.74 ± 3.75	356	2.12 ± 3.97	0.459

Data are expressed as the mean ± SD. BMI: body mass index; SBP: systolic blood pressure, DBP: diastolic blood pressure; GFR: glomerular filtration rate; K^+^: concentration of serum potassium; HbA1c: glycated hemoglobin; A/PRA: aldosterone to plasma renin activity ratio; A/PRC: aldosterone to plasma renin concentration ratio.

**Table 2:** Prevalence of positive screening for primary aldosteronism according to sex, considering both aldosterone and aldosterone–renin ratios.

	Total		Sex				*p*
			Female		Male		
	Cases/Total	% (95% CI)	Cases/Total	% (95% CI)	Cases/Total	% (95% CI)	
Aldosterone > 10 and A/PRA ≥ 30 or A/PRC ≥ 2.5	559/3464	16.1 (14.9–17.4)	297/2060	14.4 (1.9–15.9)	262/1404	18.7 (16.6–20.7)	<0.001
Aldosterone > 10 and A/PRA ≥ 30	354/2414	14.7 (13.3–16.1)	174/1366	12.7 (11.0–14.5)	180/1048	17.2 (14.9–19.5)	<0.001
Aldosterone > 10 and A/PRC ≥ 2.5	205/1050	19.5 (17.1–21.9)	123/694	17.7 (14.9–20.6)	82/356	23.0 (18.6–27.4)	0.120

Data are expressed as the mean ± SD and median; *p* = descriptive level of the Chi-square test. A/PRA: aldosterone to plasma renin activity ratio; A/PRC: aldosterone to plasma renin concentration ratio.

**Table 3:** Cutoff, sensitivity, specificity, and positive and negative predictive values (% and 95% confidence intervals) of clinical variables for confirmed primary aldosteronism.

	Cutoff Value	Sensitivity % (95% CI)	Specificity % (95% CI)	Predictive Value		Overall Accuracy ^1^ (%)	ROC (95% CI)	N
				Positive % (95% CI)	Negative % (95% CI)			
Aldosterone (ng/dL)	≥16.95	84.8 (80.0–88.8)	83.4 (82.1–84.7)	30.7 (27.4–34.1)	98.4 (97.9–98.9)	8.5	0.920 (0.908–0.933)	3464
A/PRA	≥29.88	96.2 (91.4–98.8)	90.1 (88.8–91.3)	35.9 (30.9–41.1)	99.8 (99.4–99.9)	90.4	0.963 (0.942–0.984)	2414
A/PRC	≥2.44	99.3 (96.2–100.0)	92.9 (91.1–94.5)	69.2 (62.5–75.4)	99.9 (9.3–100.0)	93.8	0.984 (0.978–0.990)	1050

^1^ Overall accuracy = percentage of correct classification.

**Table 4:** Comparison of clinical and laboratory parameters between confirmed cases (primary aldosteronism) and control groups.

	Groups		*p*
	Control	Case	
Male, number/total (%)	40.5%	40.9%	0.885^a^
Age (years old)			
N, Mean ± SD	59.2 ± 13.1	54.8 ± 12	<0.001
BMI (kg/m^2^)			
N, Mean ± SD	29.68 ± 5.65	29.31 ± 4.83	0.426
SBP (mmHg)			
N, Mean ± SD	160.43 ± 30.6	147.7 ± 31.73	<0.001
DBP (mmHg)			
N, Mean ± SD	96.12 ± 17.94	90.52 ± 17.19	<0.001
Creatinine (mg/dL)			
N, Mean ± SD	1.29 ± 1.18	1.10 ± 0.54	0.021
K^+^ (mEq/L)			
N, Mean ± SD	4.29 ± 0.52	3.72 ± 0.84	<0.001
Urinary Na^+^ (mEq/L)			
N, Mean ± SD	174.2 ± 82.25	160.05 ± 73.11	0.301
HbA1c (%)			
N, Mean ± SD	6.51 ± 1.53	6.55 ± 6.37	0.803
Aldosterone (ng/dL)			
N, Mean ± SD	11.19 ± 10.48	39.57 ± 38.67	<0.001
A/PRA			
N, Mean ± SD	12.64 ± 18.04	100.13 ± 101.58	<0.001
A/PRC			
N, Mean ± SD	0.81 ± 1.29	8.46 ± 6.75	<0.001

BMI: body mass index; SBP: systolic blood pressure, DBP: diastolic blood pressure; K^+^: concentration of serum potassium; HbA1c: glycated hemoglobin; A/PRA: aldosterone to plasma renin activity ratio; A/PRC: aldosterone to plasma renin concentration ratio.

**Table 5:** Adjusted odds ratio of potassium, aldosterone, and aldosterone–renin ratios in predicting primary aldosteronism.

	Final Model	
	Adjusted Odds Ratio (95% CI)	*p*
K^+^ (mEq/L)	0.55 (0.39–0.77)	<0.001
Aldosterone ≥ 16.95 ng/dL (ROC)	6.24 (3.97–9.81)	<0.001
A/PRA ≥ 29.88 or A/PRC ≥ 2.44	216.17 (86.34–541.22)	<0.001

A/PRA: aldosterone to plasma renin activity ratio; A/PRC: aldosterone to plasma renin concentration ratio.

## Data Availability

The data that support the findings of this study are available from the corresponding author (H.F.L.) upon reasonable request.
